# Evaluation of piperacillin-tazobactam disks using contemporary Enterobacterales isolates suggests the need for disk potency optimization

**DOI:** 10.1128/jcm.01599-24

**Published:** 2025-01-10

**Authors:** Ayesha Khan, Carmila Manuel, Richard Maynard, Romney M. Humphries

**Affiliations:** 1 Department of Pathology, Microbiology and Immunology, Vanderbilt University Medical Center 204907 , Nashville, Tennessee, USA; Cleveland Clinic, Cleveland, Ohio, USA

**Keywords:** susceptibility testing, antibiotic resistance, *Enterobacteriaceae*, diagnostics, antimicrobial agents, Gram-negative bacteria, antimicrobial activity, breakpoints

## Abstract

Piperacillin-tazobactam (TZP) is a commonly used broad-spectrum agent. OXA-1 β-lactamases drive global Enterobacterales TZP resistance and raise MICs to the clinical breakpoints (8/4–16/4 µg/mL), making susceptibility testing challenging. Two TZP disks are used globally. The first with 100 µg piperacillin and 10 µg tazobactam, established in 1992, is used by laboratories following U.S. FDA and Clinical and Laboratory Standards Institute (CLSI) standards. The second, with 30 µg piperacillin and 6 µg tazobactam, was developed by the European Committee on Antimicrobial Susceptibility Testing (EUCAST). When CLSI updated Enterobacterales TZP MIC breakpoints in 2022, it became apparent that the 100/10 µg disk may not accurately predict TZP resistance or susceptibility. In this study, we performed a disk mass titration study using 100 contemporary Enterobacterales isolates, including 40 harboring bla_OXA-1_. Relative to reference broth microdilution with CLSI breakpoints, categorical agreement (CA) for the 100/10 µg disk was 68% with 0.6% major errors (MEs) and 32% minor errors. The CA for the 30/6 µg disk against EUCAST breakpoints was 88% with 15% very major errors and 10% ME. A third disk developed in this study, 20/5 µg, yielded 81% CA with CLSI MIC breakpoints and disk breakpoints generated using the error-rate-bounded method. CA was 62.4%, 63.2%, and 70.9% for isolates with bla_OXA-1_ using the 100/10, 30/6, and 20/5 µg disks, respectively, whereas it was 71.6%, 86.9%, and 83.1% for isolates without bla_OXA-1_. Decreasing TZP disk potency improved the overall performance of disk diffusion and improved separation between susceptible and non-susceptible isolates, particularly those harboring OXA-1, but no disk yielded optimal results.

**IMPORTANCE:**

In this article, we address major gaps in contemporary data for piperacillin-tazobactam (TZP) susceptibility testing and evaluate the performance of disk diffusion. TZP is the most common empiric broad-spectrum agent against Gram-negative pathogens and is used as a carbapenem-sparing regimen. OXA-1 β-lactamases drive global Enterobacterales TZP resistance and raise MICs to the clinical breakpoints, making susceptibility testing challenging. In 2022, CLSI revised the Enterobacterales TZP MIC breakpoints. Due to the lack of contemporary correlates, disk diffusion breakpoints were revised using outdated historical data from 1991 and 2003 and yielded unacceptable error rates. Additionally, there is a lack of global consensus on disk potency. The EUCAST TZP disks contain 30 μg piperacillin and 6 μg tazobactam. The CLSI disks, established after TZP approval in 1979 and prior to the widespread prevalence of ESBLs, contain 100 μg piperacillin and 10 μg tazobactam. Here, we evaluate disk diffusion using the 100/10 and 30/6 μg TZP disks with 100 contemporary Enterobacterales isolates, including 40 harboring bla_OXA-1_. We conducted a disk development study to determine if an alternative potency might address accuracy issues with TZP susceptibility testing. We demonstrate that decreasing TZP potency improves the performance of disk diffusion and improves separation between susceptible and non-susceptible isolates, particularly those harboring OXA-1, but no disk yielded optimal results. The alternative 20/5 μg disk yielded the lowest errors using CLSI MIC breakpoints and the best separation between susceptible isolates and isolates harboring bla_OXA-1_. Our study addresses an unmet need, shows that further optimization of the TZP disk potency is possible, and provides clinical laboratories with a better understanding of the performance of TZP disks using contemporary, challenging isolates. A larger, multicenter study is needed for further optimization but has been limited by a lack of funding for an off-patent antimicrobial. Our struggles in accessing funding underscore the frequent challenge with AST for older but heavily used antimicrobials.

## INTRODUCTION

Piperacillin-tazobactam (TZP) is the most frequently utilized empiric antimicrobial agent for broad-spectrum coverage of Gram-negative bacteria and is often used as a carbapenem-sparing regimen to help mitigate the rapidly rising incidence of carbapenem resistance (1). In 2022, the Clinical and Laboratory Standards Institute (CLSI) revised TZP clinical breakpoints for Enterobacterales, to ≤8/4 µg/mL for susceptible, 16/4 µg/mL for susceptible dose-dependent (SDD), and ≥32/4 µg/mL for resistant (R) (2). The breakpoints were revised largely due to the findings of a large randomized controlled trial (MERINO) where TZP did not meet non-inferiority criteria compared to meropenem for the treatment of bloodstream infections caused by ceftriaxone nonsusceptible *Escherichia coli* and *Klebsiella pneumoniae* (3). Notably, the MERINO trial demonstrated a significantly increased risk of mortality for patients treated with TZP when the isolates yielded TZP MICs ≥ 32/4 µg/mL (4). Additionally, over the last decade, several studies have demonstrated a poor probability of pharmacokinetic/pharmacodynamic (PK/PD) target attainment (greater than 50% time above the MIC) for TZP in isolates with an MIC of 16/4 µg/mL or above (2). Thus, the revised CLSI SDD breakpoint for TZP is based on extended-infusion dosing (4.5 g every 6 h as a 3-h infusion or 4.5 g every 8 h as a 4-h infusion) that is predicted to achieve the PK/PD target of 50% time above MIC (5). Due to the lack of contemporary data on disk-to-MIC correlates, CLSI revised the TZP disk diffusion (DD) breakpoints for Enterobacterales based on historical data sets from 1992 and 2004 (6). Disk-to-MIC correlates for these revised DD breakpoints did not meet CLSI M23 error-rate acceptance criteria, yielding high rates of minor errors (MIs) and very major errors (VMEs, false susceptibility). The VME rate for isolates with MICs ≥ 64/4 µg/mL (≥I  +  2 MIC range category) was 2.2%, above the 2% acceptable rate. The MI rate was 13.4% for isolates with MICs ≥ 64/4  µg/mL and 12.4% for isolates with MICs ≤ 2/4  µg/mL, above the 5% acceptable rate, demonstrating both overcalling and undercalling of susceptible results (6). Despite these limitations, CLSI published the breakpoints in the absence of other data, as many laboratories utilize disk diffusion.

The TZP clinical breakpoints against *Pseudomonas aeruginosa* were also revised by CLSI in January 2023. Contemporary European Committee on Antimicrobial Susceptibility Testing (EUCAST) data determined that the epidemiological cutoff value for TZP against *P. aeruginosa* was ≤16/4 µg/mL. A review of PK/PD studies showed that PK/PD target attainment for MICs of 16/4 µg/mL is unlikely with standard infusion but possible with extended-infusion TZP (2). Furthermore, clinical outcome data indicated that MICs ≥ 32/4 µg/mL were associated with increased mortality rates for infections caused by *P. aeruginosa*. Thus, the TZP MIC breakpoints were updated to ≤16/4 µg/mL for susceptible, 32/4 µg/mL for intermediate, and ≥64/4 µg/mL for resistant (7). Disk-to-MIC correlate data from the 2012 *P*. *aeruginosa* TZP breakpoint revision were reanalyzed with the 2023 breakpoints. Disks tended to overcall isolates that were susceptible by broth microdilution (BMD) as intermediate and undercall isolates that were resistant by BMD as intermediate (7). Thus, the susceptible zone diameter was revised from >21 to >22 mm to reduce the likelihood of isolates with MICs of 32/4 µg/mL being miscategorized as susceptible.

Globally, many clinical laboratories, particularly those serving marginalized communities and based in under-resourced settings, rely on DD as the primary method for susceptibility testing. As such, limitations with disk diffusion correlates contribute to a lack of global accuracy in TZP testing. Furthermore, there is a lack of global consensus on disk potency. TZP disks utilized by the European Committee on Antimicrobial Susceptibility Testing contain 30 µg of piperacillin and 6 µg of tazobactam, whereas disks utilized by CLSI contain 100 µg of piperacillin and 10 µg of tazobactam (5, 8).

TZP susceptibility testing is challenging, in general, and even the reference broth microdilution method displays high variability (9, 10). In 2011, the US FDA recalled TZP on cards for a commercial automated system, Vitek2, and soon after in 2015, EUCAST released a warning statement about the poor performance of TZP gradient strips as the FDA also recalled the TZP Etest (bioMerieux, Durham, NC, USA) strip (10). These tests have since been reformulated with improved performance (11, 12). Notably, TZP susceptibility testing is particularly problematic for isolates that co-harbor extended-spectrum beta-lactamases (ESBLs) and OXA-1, a penicillinase with weak affinity for beta-lactamase inhibitors (4, 9, 13). Co-carriage of OXA-1 with an ESBL increases the TZP modal MIC from 2/4 µg/mL (in the absence of OXA-1) to 8/4–16/4 µg/mL, which straddles the S/SDD breakpoint (4, 13). Additionally, a *post hoc* analysis of 320/379 bloodstream isolates from the MERINO trial showed that isolates co-harboring OXA-1 and ESBLs were most often not susceptible by BMD but tested susceptible by other routine, commercial automated, and manual laboratory methods, including DD (4). These isolates typically had MICs of 8/4–32/4 µg/mL. A more recent study showed that while BMD reproducibility was particularly low for isolates with the *bla*_OXA-1_ resistance mechanism, the ETEST demonstrated good sensitivity in ruling out the presence of the *bla*_OXA-1_ gene in ceftriaxone non-susceptible *Enterobacterales,* although it did not necessarily agree with BMD results (9).

In this study, we aim to address the gap in contemporary TZP disk-to-MIC correlation data by evaluating the performance of the disk diffusion method, using the 100/10 µg CLSI and 30/6 µg EUCAST recommended TZP disk potencies, relative to the modal MICs from the reference BMD method against a contemporary cohort of clinical *E. coli* and *K. pneumoniae* isolates enriched for strains harboring *bla*_OXA-1_. Performance was evaluated against CLSI or EUCAST breakpoints (Table 1). Additionally, we performed a preliminary disk development study according to the CLSI M23S guidelines to determine if an alternative disk potency might address some of the accuracy issues with TZP susceptibility testing described thus far, using the same set of contemporary clinical *Enterobacterales* isolates (14).

**TABLE 1:** MIC and zone diameter *Enterobacterales* clinical breakpoints utilized for piperacillin-tazobactam

Antimicrobial agent	Disk content (µg)	Interpretive categories and zone diameter breakpoints, nearest whole (mm)					Interpretive categories and MIC breakpoints (μg/mL)				
		S	SDD	I	R	ATU	S	SDD	I	R	ATU
Piperacillin-tazobactam CLSI breakpoints	100/10	≥25	21–24	–^*a*^	≤20	–	≤8/4	16/4	–	≥32/4	–
Piperacillin-tazobactam EUCAST breakpoints	30/6	≥20	–	-	<20	19	≤8/4	–	–	>8/4	16

^*a*^ –, not applicable.

## MATERIALS AND METHODS

### Study design

**Fig 1:**
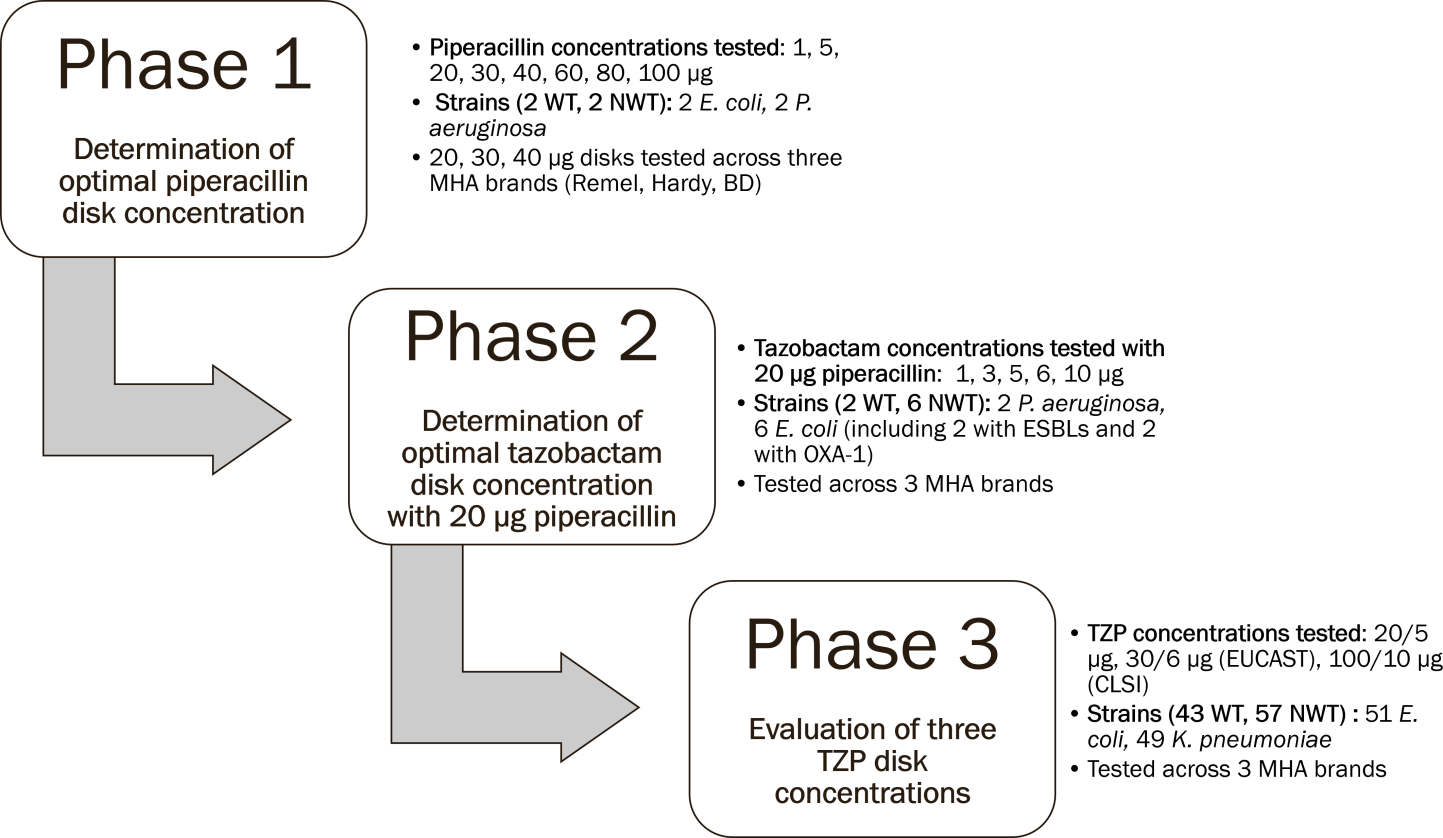
Study design.

The three phases of our study are briefly summarized in Fig. 1 and were designed according to CLSI M23 guidelines (14). In phase 1, we determined the optimal disk content of piperacillin alone by testing a range of eight potencies (1–100 µg) against four representative *E. coli* and *P. aeruginosa* strains. Three of the best-performing piperacillin disk potencies (20, 30, and 40 µg) were tested in duplicate against the same four strains across three commercial brands of Mueller Hinton agar (MHA; BD, Remel, Hardy). We then chose to proceed with the piperacillin disk content that yielded the largest zone diameter difference between wild-type (WT, defined as MIC of ≤4 µg/mL for Enterobacterales and ≤16 µg/mL for *P. aeruginosa*) and non-wild-type (NWT) strains.

In phase 2, we determined the optimal disk content of tazobactam by testing a range of five potencies (1–10 µg) alongside a fixed potency of 20 µg piperacillin against eight representative *E. coli* and *P. aeruginosa* strains. Each strain was tested in duplicate across three brands of MHA. We chose to move forward with the TZP disk potency that yielded the largest average zone diameter difference between WT and NWT strains across all brands of MHA. In the final phase 3, we used a cohort of 100 clinical *E. coli* and *K. pneumoniae* strains to evaluate the performance of three TZP disks: the disk potency optimized in our study (20 µg piperacillin/5 µg tazobactam), the CLSI disk potency (100/10 µg), and the EUCAST disk potency (30/6 µg). Each disk potency was tested across all three MHA brands yielding a total of 300 data points. Results from disk diffusion were compared to reference broth microdilution.

### Isolate collection

The isolates used in phase 1 of this study consisted of three quality control strains, *E. coli ATCC* 25922 (WT, for TZP, MIC 2/4 µg/mL), *E. coli ATCC* 35218 (NWT, for TZP, MIC ≥ 64/4 µg/mL), *P. aeruginosa ATCC*27853 (WT, MIC 4/4 µg/mL), and one NWT *P. aeruginosa* clinical bloodstream isolate from our institution (MIC ≥ 64/4 µg/mL). The isolates used in phase 2 included these four isolates in addition to four NWT *E. coli* clinical isolates (MICs ≥ 64/4 µg/mL). In phase 3, 100 isolates were evaluated across three MHA brands: 2 *E. coli* ATCC (25922 and 35218) strains and 98 *E. coli* (*n* = 49) and *K. pneumoniae* (*n* = 49) strains isolated from clinical specimens in 2022 as part of the SENTRY Antimicrobial Surveillance Program (JMI Labs). Isolates with a diverse distribution of MICs were selected (43 WT and 57 NWT isolates). These isolates were described previously, including ESBL and bla_OXA-1_ gene content (9). Isolate and MIC distributions, based on modal TZP MICs and the presence or absence of *bla*_OXA-1_, are shown in Table 2. Each isolate was subcultured twice from the frozen stock onto Sheep’s Blood Agar (BD, Sparks, MD, USA) prior to testing by BMD and DD.

**TABLE 2:** Distribution of clinical bacterial isolates used to assess the performance of TZP disks in phase 3, with modal MIC determined by triplicate reference broth microdilution^*a*^

Species	*bla*_OXA-1_		Number of isolates at MIC				
		Total	MIC ≤ 4/4 µg/mL	MIC 8/4 µg/mL (S breakpoint)	MIC 16/4 µg/mL (SDD)	MIC 32/4 µg/mL (R breakpoint)	MIC ≥ 64/4 µg/mL
*E. coli*	Absent	30	22	4	3	0	1
	Present	21	0	4	4	4	9
	Total	51	22	8	7	4	10
*K. pneumoniae*	Absent	30	21	3	3	0	3
	Present	19	0	4	4	7	4
	Total	49	21	7	7	7	7

^*a*^ S, susceptible; SDD, susceptible dose dependent; R, resistant.

### Broth microdilution testing

BMD was performed according to CLSI guidelines and has been described previously (9). Briefly, 96-well panels were prepared in-house with the HTF plate dispenser system (MDZ Automation, Chicago, IL, USA). Each panel included a range of piperacillin concentrations (128–2 µg/mL) in a twofold dilution series in cation-adjusted Mueller-Hinton broth (CA-MHB, BD) with the tazobactam concentration fixed at 4 µg/mL. Piperacillin and tazobactam were obtained from US Pharmacopeia (Rockville, MD, USA). A single lot of panels stored at −70°C was used for the whole study. *E. coli* ATCC 25922, *E. coli* ATCC 35218, and *P. aeruginosa* ATCC 27853 were used for quality control each day of testing. A 0.5 McFarland standard was prepared in sterile water using fresh subcultures and diluted to inoculate the BMD panels according to the M07 guidelines (15). Plates were incubated at 35°C in ambient air for 16–18 h and read independently by two readers.

### Disk diffusion testing

Disk diffusion was performed according to the CLSI M02 guidelines with M23 procedures to establish optimal TZP disk potencies (14, 15). Briefly, disks were manually prepared in-house by impregnating paper disks with various concentrations of piperacillin and/or tazobactam. As per M100 guidelines, stock solutions were prepared at 50 times the final highest disk potency (5). Diluted working solutions were prepared as needed such that 20 µL was added to a paper disk in a petri dish to achieve the final desired potency. Disks were dried and stored in a sterile container with desiccant at 2°C–8°C, until use. Disks were used within 2 weeks of preparation following M23 guidelines. The potency and shelf life of the manually prepared disks were verified by testing QC strains in triplicate immediately after preparation and alongside all subsequent testing.

### Data analysis

Disk diffusion results were compared to modal MICs generated from six replicates by reference BMD (9). Phase 3 of the study tested 100 isolates across three brands of MHA, yielding a total of 300 data points for analysis. Categorical agreement (CA), VMEs, major errors (MEs), and MIs were evaluated using the error rate-bound method (14). CA is defined as the agreement of interpretive results between the disk diffusion and reference BMD using CLSI or EUCAST breakpoints. Breakpoints evaluated in this study are in Table 1. Best fit disk diffusion breakpoint estimates were generated when appropriate with disk-to-MIC correlates on the diffusion Breakpoint Estimation Testing Software (dBETS, version 1.5; https://dbets.shinyapps.io/dBETS/). Discordant results between disk diffusion and BMD were categorized as follows: VME, false-susceptible result for an isolate that is resistant by BMD; ME, false-resistant result for an isolate that is susceptible by BMD; and MI, a discrepancy between disk diffusion and BMD involving a susceptible dose-dependent result. VME rates were determined using the number of isolates that were resistant by BMD as the denominator, ME rates were determined using the number of isolates that were susceptible by BMD as the denominator, and MI rates were determined using the total number of isolates as the denominator. Acceptable VME, ME, and MI error rates for isolates with MICs of 8/4–32/4 µg/mL (i.e., BMD MIC values within 1 doubling dilution of the SDD breakpoint) were <10%, <10%, and <40%, respectively. For isolates with MICs ≤ 4/4 or >64/4 µg/mL (i.e., BMD MIC values outside 1 doubling dilution of the SDD breakpoint), acceptable VME, ME, and MI error rates were <2%, <2%, and <5%, respectively.

## RESULTS

### Phase I: selection of optimal piperacillin disk potency

In Phase 1 of the study, we sought to determine the optimal potency of piperacillin for the TZP combination disk. Disks with a range of eight different piperacillin potencies (1, 5, 20, 30, 40, 60, 80, and 100 µg) were tested simultaneously alongside the reference piperacillin BMD method against four representative wild-type (BMD MIC 4 µg/mL) and non-wild-type (BMD MICs ≥ 64 µg/mL) *E. coli* and *P. aeruginosa* strains. Disks were evaluated against M23-described optimal zone diameters and the zone of growth inhibition difference between WT and NWT isolates (14). Disks with 20–100 μg piperacillin exhibited zone diameters between 23 and 28 mm, which met the M23 criteria (zone diameters should ideally not be above 30 mm). The 20 µg piperacillin disk yielded the largest difference in zone diameters between WT and NWT strains (12 mm difference for *E. coli* and 17 mm difference for *P. aeruginosa*) followed by the disks with 30 and 40 µg of piperacillin, which yielded the same results (Table S1).

The best-performing three disk potencies, based on zones of growth inhibition ≤30 mm and the largest difference in zone size between WT and NWT, were 20, 30, and 40 µg of piperacillin. These were subsequently tested in replicate against the same four WT and NWT strains across three brands of MHA(Remel, Hardy, and BD), and the results were compared to the BMD MICs (Table S1). The disks performed similarly across all three brands of MHA. The 20 µg piperacillin disk yielded the largest average zone diameter difference between WT and NWT strains across the three brands of MHA (11 mm for *E. coli* and 16 mm for *P. aeruginosa*) followed by the 30 µg disk (10.7 mm for *E. coli* and 15 for *P. aeruginosa*) and the 40 µg disk (11 mm for *E. coli* and 13.2 for *P. aeruginosa*). Thus, 20 µg was chosen as the optimal piperacillin potency for phase 2 of the TZP disk development study.

### Phase 2: selection of optimal tazobactam potency for piperacillin-tazobactam disk

In phase 2, we screened disks containing five different potencies of tazobactam (1, 3, 5, 6, and 10 µg) in combination with a fixed potency of piperacillin (20 µg) against two WT *E. coli,* four NWT *E. coli,* one WT *P. aeruginosa,* and one NWT *P. aeruginosa* isolate and compared the results to modal TZP BMD MICs (Table S3). Of the clinical *E. coli* isolates, one with an elevated TZP MIC of 8/4 µg/mL harbored *bla*_TEM-1_, one resistant isolate (MIC >64/4 µg/mL) harbored *bla*_CTX-M-15,_ and two strains, with MICs of 16/4 µg/mL (SDD) and ≥64/4 µg/mL, co-harbored *bla*_OXA-1_. The disk with 20 µg of piperacillin and 5 µg of tazobactam yielded the largest average zone diameter difference between all the WT and NWT strains (9.33 mm for *Enterobacterales* and 14.67 for *P. aeruginosa*) across all three brands of MHA (Table S3). As such, the 20/5 µg TZP disk was chosen for further performance evaluation.

### Phase 3: comparison of 100/10 μg (CLSI) and 30/6 μg (EUCAST) TZP disks to the 20/5-optimized disk

**Fig 2:**
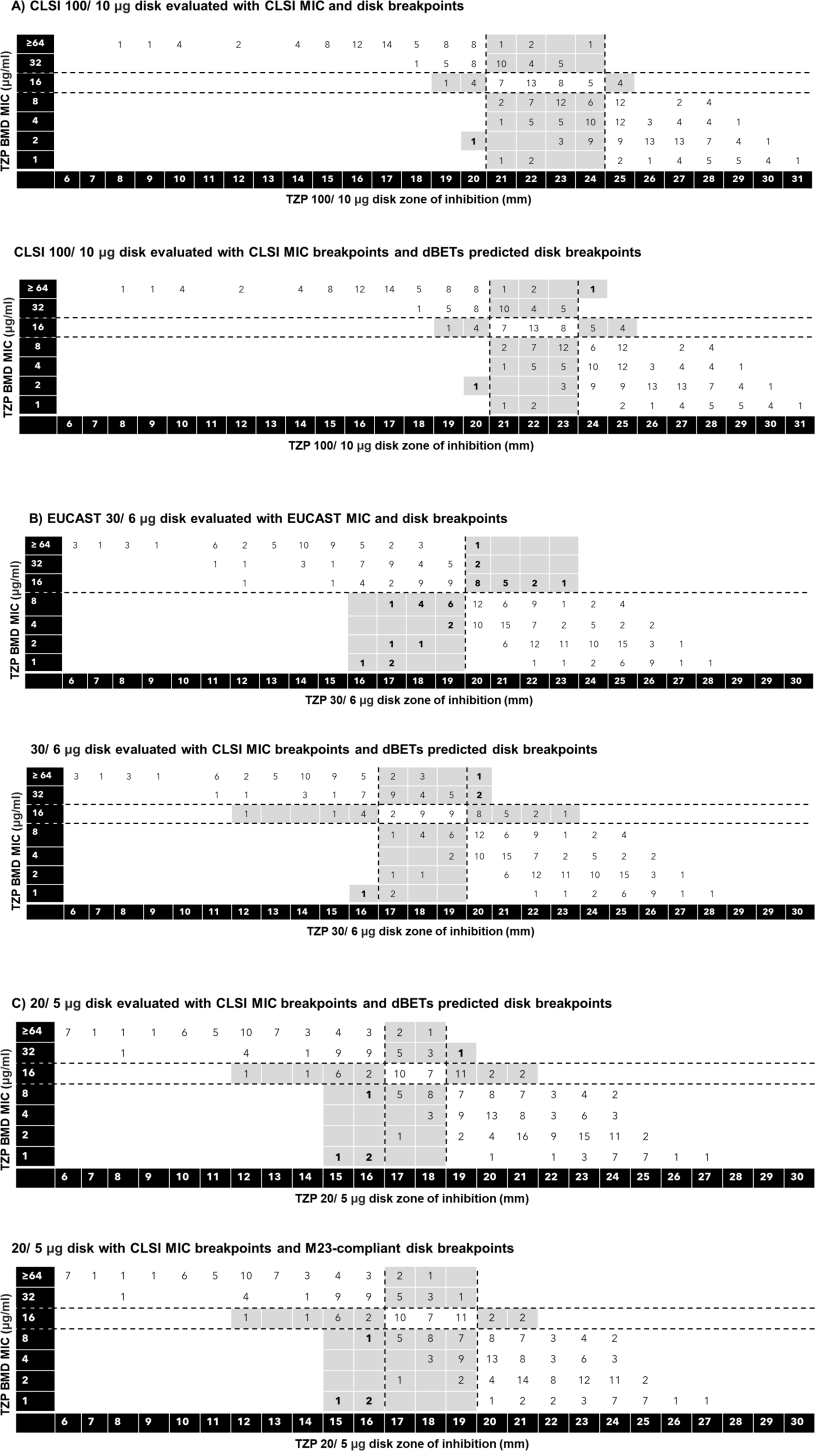
Correlation between BMD MIC values and disk diffusion zone diameters determined using (A) the100/10 µg CLSI TZP disk with CLSI MIC breakpoints and CLSI disk breakpoints or dBETs predicted best fit error-rate-bounded disk breakpoints; (B) the 30/6 µg EUCAST disk with EUCAST MIC breakpoints and EUCAST disk breakpoints or dBETs predicted error-rate-bounded disk breakpoints; and (C) the 20/5 µg TZP disk with CLSI MIC breakpoints and dBETs program predicted disk breakpoints by error-rate-bound method or manually selected disk breakpoints (16 and 20 mm) with an M23-compliant intermediate zone. Errors are shaded. Very major and major errors are bolded.

A total of 100 *E. coli* and *K. pneumoniae* isolates were tested by BMD and DD with the disks under evaluation. Forty isolates harbored *bla*_OXA-1_ (Table 2). In the cohort, 58 isolates were susceptible by CLSI TZP breakpoints and 14 were SDD and 28 were resistant by reference BMD (Table 2). Forty isolates had BMD MICs ± 1 doubling dilution from the clinical breakpoints (8/4, 16/4, or 32/4 µg/mL). DD and BMD MIC results for the CLSI recommended disk potency (100/10 µg) are displayed in Fig. 2A for the EUCAST disk (30/6 µg) in Fig. 2B and this study’s 20/5 µg disk in Fig. 2C. Error rates for all three disks were evaluated using reference MIC values (Table S4).

When CLSI breakpoints were applied to BMD MICs and zone diameters derived from the 100/10 µg disk (Fig. 2A), the overall CA was 68% (204/300) with 1 ME (0.6%) and 95 MIN (31.7%). Of the 95 MIN, 55 (45.8%) were within one doubling dilution of the intermediate breakpoint (I *±* 1 category), 36 (27.1%) were due to overcalling (≤I-2 category), and 4 isolates (5.6%) were due to undercalling (≥I + 2 category) resistance (Table S4). The MIN rate for isolates with MIC ≤ 4/4 µg/mL was well above the M23 acceptable limit of 5%, and the MIN rate for isolates with MIC ≥ 64/4 µg/mL was slightly above acceptable (Table S4). The MIC values and DD results from the 100/10 µg disk were also evaluated by the dBETs program to determine if an alternative disk-to-MIC correlate would improve performance for the CLSI 100/10 disk, using the error-rate-bounded method (Fig. 2A). The dBETs software determined that the optimal disk breakpoints were ≥24 mm for susceptibility and ≤20 mm for resistance (1 mm difference from the CLSI-defined ≥25 mm susceptible and ≤20 mm resistant breakpoints). Evaluation of the data with these revised breakpoints increased the CA to 74.7% (224/300), reduced the number of MIN to 74 (24.7%), and yielded one VME (1%) and one ME (0.6%) (Table S4). Of note, the MIN rate for isolates in the ≤4/4 µg/mL category was 17%, which is above M23 acceptance limits of 5%.

When EUCAST breakpoints were applied to MICs and zone diameters derived from the 30/6 µg disk (Fig. 2B), overall CA was 87.7% (263/300), with 19 VME (15.1%) and 18 ME (10.3%). There is no intermediate or SDD category with the EUCAST breakpoints, so MIs are not calculated. Of the 19 VME, 16 (18.3%) were at the susceptible or resistant breakpoint (MIC, 8/4–16/4 µg/mL) and 3 (3.6%) were above the resistant breakpoint (MIC ≥ 32/4 µg/mL). Of the 18 ME, 11 (12.6%) were at the susceptible or resistant breakpoint (8/4–16/4 µg/mL) and 7 (5.4%) were below the susceptible breakpoint (≤4/4 µg/mL, Table S4). The VME and ME rates are above the M23 acceptable limits, which are <10% in the 8/4–16/4 µg/mL category and <2% for the ≥32/4 µg/mL or ≤4/4 µg/mL categories. The error-rate-bounded method was used to determine disk-to-MIC correlates with the CLSI TZP MIC breakpoints and the 30/6 disk. This analysis yielded optimal disk breakpoints of ≥20 mm for susceptible and ≤16 mm for resistant (Fig. 2B). These revised breakpoints yielded 78% (234/300) CA with 3 VME (3.5%), 1 ME (0.6%), and 62 MIN (20.7%), with reference BMD MIC results (Table S4). While the VME and ME rates were within acceptance limits as defined by CLSI M23, the MIN rate was just above the acceptable criteria for isolates in the 4/4–32/4 µg/mL (42.5% vs acceptable ≤40%) and unacceptable for isolates in the ≥64/4 µg/mL category (9.8% vs acceptable ≤5%).

Finally, disk correlates were established for the 20/5 µg disk by the error-rate-bounded method. This analysis yielded optimal disk breakpoints of ≥19 mm for susceptibility and ≤16 mm for resistance (Fig. 2C). The CA with BMD was 80.7% (242/300) with 1 VME (1.2%), 4 ME (2.3%), and 53 MIN (17.7%) (Table S4). The MIN rate was slightly above acceptable for isolates in the ≥64/4 µg/mL category (5.9% vs acceptable 5%). Though the error rates overall either met or were very near M23 acceptance criteria, the 17–18 mm intermediate zone is noncompliant with M23 guidelines, which require the intermediate range to equal at least half of the QC range (24–30 mm). If the susceptible breakpoint is shifted to 20 mm, making the intermediate zone M23 compliant (Fig. 2C), the CA decreases slightly to 78.3% (235/300) with an increase in MIN to 61 (20.3%), no VME, and the same four ME (2.3%, Table S4). The MIN rate is slightly above acceptable levels for isolates in the ≥64/4 µg/mL category (5.9%) and ≤4/4 µg/mL category (11.6%). Overall, the 20/5 µg disk improved the separation between the susceptible and non-susceptible clinical Enterobacterales isolates (Table 3) and yielded the largest zone diameter difference between them (6.7 mm) relative to the 30/6 µg disk (6.4 mm) and 100/10 µg disk (5.7 mm).

**TABLE 3:** Average disk diffusion zone diameters (mm) with the three disk potencies (20/5, 30/6, and 100/10 µg) parsed by susceptibility categories using TZP CLSI breakpoints or based on the presence of *bla*_OXA-1_^*c*^

	*N*	20/5 µg	30/6 µg	100/10 µg
S	174	21.3	22.4	25.4
SDD	42	17.5	18.8	22.3
R	96	13.1	14.6	18.4
NS	138	14.6	16	19.7
Δ S-R^*d*^	–	8.2	7.8	7.0
Δ S-SDD	–	3.8	3.6	3.1
Δ S-NS	–	6.7	6.4	5.7
MIC ≤ 4/4	129	21.8	22.9	25.8
MIC ≥ 8/4 with bla_OXA-1_	117	15.3	16.7	20.4
MIC ≥ 8/4 without bla_OXA-1_	183	17.4	18.5	21.8
^*a*^Δ with bla_OXA-1_	–	6.5	6.2	5.4
^*b*^Δ without bla_OXA-1_	–	4.4	4.4	4.0

^*a*^ Δ with *bla*_OXA-1_ is the average zone diameter difference between isolates with MICs ≤ 4/4 µg/mL and isolates with MICs ≥ 8/4 µg/mL that harbor *bla*_OXA-1_.

^*b*^ Δ without *bla*_OXA-1_ is the average zone diameter difference between isolates with MICs ≤ 4/4 µg/mL and isolates with MICs ≥ 8/4 µg/mL that do not harbor *bla*_OXA-1_.

^*c*^ Δ indicates the average difference between the zone diameters of the strains indicated.

^*d*^ –, not applicable.

### Disk performance compared to the presence of OXA-1

We noted a difference in performance for isolates with and without *bla*_OXA-1_, as expected. Overall CA was 62.4%, 63.2%, and 70.9% for isolates with *bla*_OXA-1_ using the 100/10, 30/6, and 20/5 µg disk potencies, respectively, whereas it was 71.6%, 86.9%, and 83.1% for isolates without *bla*_OXA-1_ (Table 4). As has been demonstrated for other susceptibility testing methods, DD performs particularly poorly against isolates coharboring ESBL and *bla*_OXA-1_ genes (9, 11). However, this performance improves significantly with decreasing the potency of piperacillin and tazobactam in the disk. As shown in Table 3, the 20/5 µg disk yields the largest average zone diameter difference (6.5 mm) between isolates with MIC values ≤4/4 µg/mL and isolates harboring *bla*_OXA-1_ with MIC values ≥8/4 µg/mL relative to the 30/6 µg disk (6.2 mm) and the 100/10 µg disk (5.4 mm). The 20/5 and 30/6 µg disks yield the same average zone diameter difference (4.4 mm) between isolates with MIC values ≤4/4 µg/mL and isolates without *bla*_OXA-1_ with MIC values ≥8/4 µg/mL.

**TABLE 4:** Summary of CA and errors using the breakpoints indicated, based on the presence or absence of the *bla*_OXA-1_ gene

Gene	*N*	% CA	VME	%	ME	%	MIN	%
**100/10 µg disk with CLSI MIC and disk breakpoints (S ≥ 25 mm, SDD 21–24 mm, and R ≤ 20 mm)**								
*bla*_OXA-1_	117	62.4	0	0	0	0	44	37.6
No *bla*_OXA-1_	183	71.6	0	0	1	0.7	51	27.9
Total	300	68	0	0	1	0.6	95	31.7
**30/6 µg disk with EUCAST MIC and disk breakpoints (S ≥ 20 mm, R ≤ 20 mm, and ATU 19 mm)**								
*bla*_OXA-1_	117	84.6	13	11.1	5	4.3	N/A^*a*^	N/A
No *bla*_OXA-1_	183	89.6	6	3.3	13	7.1	N/A	N/A
Total	300	87.7	19	6.3	18	6	N/A	N/A
**20/5 µg disk with CLSI MIC breakpoints and dBETs predicted disk breakpoints (S ≥ 20 mm, SDD 17–19 mm, and R ≤ 16 mm)**								
*bla*_OXA-1_	117	70.9	0	0	1	4.8	33	28.2
No *bla*_OXA-1_	183	83.1	0	0	3	2	28	15.3
Total	300	78.3	0	0	4	2.3	61	20.3

^*a*^ N/A, not applicable.

## DISCUSSION

TZP has long been used as an important antimicrobial for empiric and definitive therapy of infections caused by Gram-negative pathogens. Several patients who failed therapy in the MERINO trial had infections caused by isolates that were initially incorrectly reported as susceptible to TZP by routine clinical laboratory susceptibility testing methods, and isolates coharboring ESBL and *bla*_OXA-1_ genes, with BMD MICs straddling the breakpoints (8/4–32/4 µg/mL), accounted for a significant proportion of the discordant, problematic isolates. The prevalence of the OXA-1 penicillinases, which are resistant to inhibition by tazobactam, is more widespread and concerning than when TZP disks were first developed (16). An estimated 30% of ceftriaxone non-susceptible *E. coli* and *K. pneumoniae* isolates in the United States harbor *bla*_OXA-1_ (JMI data on file), and the global prevalence of *K. pneumoniae* isolates harboring *bla*_OXA-1_ is approximately 51% (17). Thus, improvement of TZP testing methods to detect this resistance mechanism is needed.

When CLSI revised TZP breakpoints in 2021, disk diffusion correlates were established using historical disk-to-MIC data from the 1992 CLSI agenda book when TZP was first evaluated (620 isolates) along with data from a 2004 study on ESBL-producing isolates (*n* = 632) (6). A major limitation of those data is that the incidence of the OXA-1 resistance mechanism in these historical isolates is unknown, and no disk breakpoint could be developed that met all CLSI M23 acceptance criteria (3.3% VME and 17.3% MIN were found). Since the publication of the CLSI breakpoints, clinical laboratories have anecdotally reported challenges with the use of DD as a reference method for the implementation of the revised TZP breakpoints on commercial antimicrobial susceptibility testing (AST) systems. We sought to investigate if a lower disk mass may be better able to detect the subtle low-level resistance seen in many contemporary Enterobacterales. Our findings highlight the need for further optimization of TZP disk potency. In particular, the 100/10 µg disk potency failed to adequately distinguish susceptible and not-susceptible isolates, especially those harboring *bla*_OXA-1_ (Fig. 2A; Tables 3 and 4). While the EUCAST disk of 30/6 µg performed better than the 100/10 disk, errors were still above the tolerance limits defined by CLSI (Table S4). Our preliminary assessment of alternative disk potencies demonstrated that a 20/5 µg disk yielded lower errors than either the CLSI or EUCAST disk when compared to CLSI MIC breakpoints (Table S4). Furthermore, the 20/5 µg disk yielded the best separation between susceptible isolates and isolates harboring *bla*_OXA-1_ (Tables 3 and 4). However, performance still did not meet CLSI M23 criteria, and investigation of even lower piperacillin disk potencies (e.g., in the >5 to <20 µg range) may be needed. In phase 2 of the study, the 20/3 µg disk performed similarly to the 20/5 µg disk with an average zone diameter difference between the WT and NWT strains of 9.25 and 9.33 mm, respectively (Table S3). Thus, the 20/3 and 20/4 µg TZP disk potencies could also be further evaluated in the future.

The 100 µg piperacillin CLSI disk potency was established shortly after the introduction of the antimicrobial in a 1979 study, where 100, 150, and 200 µg disk potencies were evaluated against *Staphylococcus aureus* isolates (16). At the time, a 100 µg carbenicillin disk was standard for testing Enterobacterales and *Pseudomonas,* which prompted studies to assess susceptibility testing methods for novel, extended-spectrum penicillins in the same potency range (18). The subsequent TZP disk optimized the tazobactam content relative to a fixed potency of 100 µg piperacillin. Both piperacillin and TZP disk potencies were optimized prior to the widespread prevalence of ESBLs. Given today’s drastically different epidemiological landscape of resistance mechanisms, there is a dire need for the piperacillin and TZP disk potency to be re-evaluated against a diverse, contemporary collection of clinical isolates.

This study has limitations. The isolates were tested by BMD and DD using different inocula for some parts of the study (i.e., phase 3). However, appropriate QC strains were included in each run, and isolates were tested by DD across three brands of MHA using the same inocula. Furthermore, the MIC results for these isolates have been robustly evaluated across multiple lots of piperacillin, tazobactam, and CA-MHB, yielding a robust modal MIC for each isolate, based on *n* = 6 readings (9). In this study, we enriched for isolates that harbor the *bla*_OXA-1_ gene and MICs near the CLSI breakpoint, which likely inflated discordance and error rates. However, this was done purposely to evaluate the “worst-case” scenario for testing Enterobacterales against TZP, which we feel is appropriate for a disk-mass evaluation study. We used the error-rate-bounded method for evaluation, which accepts an increased error rate for isolates within one doubling dilution of the intermediate breakpoint (Table S4; Fig. 1, 2.). We only evaluated Enterobacterales, but TZP is widely used for treating other Gram-negative infections, including *P. aeruginosa*. We did not evaluate *P. aeruginosa* because at the time of this study, CLSI was deliberating TZP breakpoint updates for *P. aeruginosa*, which have since been published and we plan to conduct further studies (7). However, it is clear that the current TZP disks are likely to under-perform for *P. aeruginosa*, given the substantially better separation of WT and NWT *P. aeruginosa* with a piperacillin disk of 20 vs 100 µg (Table S1). Finally, the variable concentration of tazobactam relative to piperacillin (i.e., a fixed concentration in MIC-based testing and a fixed ratio in disk diffusion) may contribute to poor MIC-to-disk correlates, which can be explored in future studies.

We compared relative overall agreement between the three disk potencies for isolates with and without *bla*_OXA-1_ (Tables 3 and 4) and noted improvements with decreasing TZP potency independent of acceptability criteria. This study demonstrates that further optimization of the TZP disk potency is possible and provides better understanding of the performance of EUCAST and CLSI TZP disks, for clinical laboratories using challenging isolates. A larger, multicenter study is needed between CLSI and EUCAST to further optimize TZP disk diffusion but has been limited by no available funding opportunities for this work on an off-patent antimicrobial. The struggles in obtaining funding for this important work underscore the frequent challenge with AST for older but heavily used antimicrobials, which are largely driven by volunteer efforts and institutional funds.

## SUPPLEMENTAL MATERIAL

Supplemental tables jcm.01599-24-s0001.pdf Tables S1 to S4.

ASM does not own the copyrights to Supplemental Material that may be linked to, or accessed through, an article. The authors have granted ASM a non-exclusive, world-wide license to publish the Supplemental Material files. Please contact the corresponding author directly for reuse.
