# Supplementary material for: Evaluation of piperacillin-tazobactam disks using contemporary Enterobacterales isolates suggests the need for disk potency optimization
Source: J Clin Microbiol. 2025 Jan 10;63(2):e01599-24. doi: 10.1128/jcm.01599-24 (PMC11837518; doi:10.1128/jcm.01599-24)
Supplement: Supplemental tables — Tables S1 to S4. [file jcm.01599-24-s0001.pdf]

Supplementary Table 1. Wild-type (WT) and non-wild-type (NWT) disk diffusion zones of growth inhibition derived from disks with a range of piperacillin potencies.

Isolate	WT/ NWT	BMD MIC	Piperacillin disk potency Zone of Growth Inhibition (mm)							
			1 ug	5 ug	20 ug	30 ug	40 ug	60 ug	80 ug	100 ug
E. coli 25922	WT	4	6	8	23	24	25	26	26	28
E. coli 35218	NWT	> 64	6	6	11	12	13	15	16	17
**Difference in WT & NWT zone of inhibition (mm)**			**0**	**2**	**12**	**12**	**12**	**11**	**10**	**11**
P. aeruginosa 27853	WT	4	9	16	23	24	25	26	26	27
P. aeruginosa #1201	NWT	> 64	6	6	6	9	10	12	14	15
**Difference in WT & NWT zone of inhibition (mm)**			**3**	**10**	**17**	**15**	**15**	**14**	**12**	**12**

Supplementary Table 2. Wild-type (WT) and non-wild-type (NWT) isolates with piperacillin broth microdilution (BMD) and disk diffusion results with 20, 30 or 40 µg piperacillin disks performed in replicate across 3 brands of Mueller Hinton Agar (BD, Hardy, Remel). MIC values (µg/ml) and disk diffusion zone diameters (mm) are listed. Δ WT-NWT mm indicates the average difference between the zone diameters of the WT strains relative to the NWT strains.

Isolate				BD			Hardy			Remel		
		Rep	MIC	20 µg	30 µg	40 µg	20 µg	30 µg	40 µg	20 µg	30 µg	40 µg
E. coli 25922	WT	1	2	23	22	20	22	21	21	24	22	20
		2	2	24	22	20	22	21	21	23	21	21
E. coli 35218	NWT	1	> 64	12	11	10	12	11	9	12	10	9
		2	> 64	12	11	10	12	11	10	12	11	9
**Average Zone difference WT and NWT**				**11.5**	**11**	**10**	**10**	**10**	**11.5**	**11.5**	**11**	**11.5**
P. aeruginosa 27853	WT	1	4	23	21	19	23	22	20	23	21	19
		2	4	22	20	18	23	21	20	22	21	19
P. aeruginosa #1201	NWT	1	> 64	8	6	6	6	6	6	6	6	6
		2	> 64	8	6	6	6	6	6	6	6	6
**Average Zone difference WT and NWT**				**14.5**	**14.5**	**12.5**	**17**	**15.5**	**14**	**16.5**	**15**	**13**

Supplementary Table 3. Wild-type (WT) and non-wild-type (NWT) isolate disk diffusion results with varying tazobactam potency, using a 20 ug piperacillin disk. **Δ WT-NWT mm** indicates the average difference in zone diameter between the WT and NWT strains.

Isolate (WT/ NWT)	**Resistance gene**	**MIC**	**Zone of growth inhibition (mm)**														
			BD MHA					Remel MHA					Hardy MHA				
			**20/1 µg**	**20/3 µg**	**20/5 µg**	**20/6 µg**	**20/10 µg**	**20/1 µg**	**20/3 µg**	**20/5 µg**	**20/6 µg**	**20/10 µg**	**20/1 µg**	**20/3 µg**	**20/5 µg**	**20/6 µg**	**20/10 µg**
E. coli 25922 (WT)	-	2	21	21	21	22	22	21	21	21	21	21	21	21	21	21	21
E. coli 35218 (WT)	TEM-1	2	17	21	22	23	24	19	21	23	23	23	19	21	23	23	24
E. coli 854173 (NWT)	TEM-1	8	14	16	17	17	17	16	17	18	18	18	16	16	17	17	18
E. coli 1173123 (NWT)	CTXM-15	≥ 64	6	6	7	9	12	6	8	9	11	13	6	7	8	11	12
E. coli 941370 (NWT)	OXA-1, OXA-30	16	14	15	15	15	16	13	15	15	16	17	13	14	15	16	17
E. coli 1045006 (NWT)	OXA-1, OXA-30	≥ 64	6	9	10	10	10	8	9	10	10	11	8	9	9	10	11
Δ WT-NWT mm			**9**	**9.5**	**9.25**	**9.75**	**9.25**	**9.25**	**8.75**	**9**	**8.25**	**7.25**	**9.25**	**9.5**	**9.75**	**8.5**	**8**
P. aeruginosa 27853 (WT)	-	8	21	21	21	21	21	20	20	20	20	20	21	21	21	21	21
P. aeruginosa #1201 (NWT)	-	≥ 64	6	6	6	6	6	6	6	6	6	6	6	6	6	6	6
Δ WT-NWT mm			**15**	**15**	**15**	**15**	**15**	**14**	**14**	**14**	**14**	**14**	**15**	**15**	**15**	**15**	**15**

Supplementary Table 4. Piperacillin-tazobactam disk diffusion results compared to reference broth microdilution interpreted using CLSI, EUCAST or dBETs software generated disk breakpoints (BPs). Error rates above M23 acceptance criteria are **bolded.**

MIC range (µg/mL)	Number	CA (%)[a]	VME (%)[b]	ME (%)[c]	MI (%)[d]
100/ 10 µg disk evaluated with CLSI MIC and disk BPs (S ≥25 mm, SDD 21-24 mm, R ≤20 mm)					
≥64/4	71	-	0 (0.0)	N/A	**4 (5.6)**
8/4-32/4	120	-	0	0	**55 (45.8)**
≤4/4	130	-	NA	1 (0.8)	**36 (27.7)**
Total	300	204 (68)	0	1 (0.6)	**95 (31.7)**
100/ 10 µg disk evaluated with CLSI MIC BPs and dBETs predicted disk BPs (S ≥24 mm, SDD 21-23 mm, R ≤20 mm)					
≥64/4	71	-	1 (1.4)	N/A	3 (4.2)
8/4-32/4	120	-	0	0	54 (45)
≤4/4	130	-	NA	1 (0.8)	**17 (13.1)**
Total	300	224 (74.7)	1(1)	1 (0.6)	**74 (24.7)**
30/ 6 µg disk evaluated with EUCAST MIC and disk BPs (S ≥20 mm, R ≤20 mm, ATU 19 mm)					
≥32/4	84	-	**3 (3.6)**	NA	**-**
8/4-16/4	87	-	**16 (18.3)**	**11 (12.6)**	-
≤4/4	129	-	NA	**7 (5.4)**	-
Total	300	263 (87.7)	19 (15.1)	18 (10.3)	**-**
30/ 6 µg disk evaluated with CLSI MIC BPs and dBETs predicted disk BPs (S ≥20 mm, SDD 17-19 mm, R ≤16 mm)					
≥64/4	51	-	1 (1.9)	NA	**5 (9.8)**
8/4-32/4	120	-	2 (1.7)	0	**51 (42.5)**
≤4/4	129	-	NA	1 (0.8)	6 (4.7)
Total	300	234 (78)	**3 (3.5)**	1 (0.6)	**62 (20.7)**
20/ 5 µg disk evaluated with CLSI MIC BPs and dBETs predicted disk BPs (S ≥19 mm, SDD 17-18 mm, R ≤16 mm)					
≥64/4	51	-	0	NA	**3 (5.9)**
8/4-32/4	120	-	1 (0.8)	1 (0.8)	46 (38.3)
≤4/4	129	-	NA	3 (2.4)	4 (3.1)
Total	300	242 (80.7)	1 (1.2)	4 (2.3)	**53 (17.7)**
20/ 5 µg disk evaluated with CLSI MIC BPs and M23-compliant disk BPs (S ≥20 mm, SDD 17-19 mm, R ≤16 mm)					
≥64/4	51	-	0	NA	**3 (5.9)**

8/4-32/4	120	-	0	1 (0.8)	43 (35.8)
≤4/4	129	-	NA	3 (2.3)	**15 (11.6)**
Total	300	235 (78.3)	0 (0.0)	4 (2.3)	**61 (20.3)**

[a]Number of isolates in CA/total isolates, [b]Number of VME/resistant isolates, [c]Number of ME/susceptible isolates, [d]Number of MIN/total isolates.

Susceptible (S), Susceptible Dose-Dependent (SDD), Resistant (R), Area of Technical Uncertainty (ATU)
